# Condition the water before adding the fish: a hypothesis on probiotic selection without prior gut assessment

**DOI:** 10.3389/fmicb.2026.1931519

**Published:** 2026-08-26

**Authors:** Puiman Chun, Xiaoli Wu, Shan Liang, Feng Jin

**Affiliations:** 1 Key Laboratory of Mental Health, Institute of Psychology, Chinese Academy of Sciences, Beijing, China; 2 Mirai Food Academic Institute of Japan, Akita, Japan

**Keywords:** autism spectrum disorder, condition-then-attract paradigm, gut microbiome assessment, gut–brain axis, metabolic potency, probiotic selection

## Abstract

**Introduction:**

Current clinical practice often assumes that gut microbiome assessment before selecting probiotic species, assuming a ‘test-then-match’ strategy yields better outcomes. However, routine testing has limited predictive value for probiotic efficacy, and many commercial probiotics fail to achieve sustained clinical improvement.

**Methods:**

In this Hypothesis and Theory article, we propose a novel, testable hypothesis: prioritize remodelling the gut environment using metabolically potent probiotics, (i.e., strains with high capacity to produce beneficial metabolites such as short-chain fatty acids, bioactive peptides, and organic acids) without prior microbiome assessment—a ‘condition-then-attract’ strategy. Our hypothesis is motivated by preclinical studies demonstrating the metabolic potency of specific strains of *Lactobacillus helveticus* and *Lactobacillus fermentum* (e.g., NS8 and NS9 originally isolated from Mongolian fermented koumiss).

**Results:**

We derive several directly testable predictions from this framework, including that a randomized controlled trial comparing ‘test-then-match’ versus ‘condition-then-attract’ strategies would show that prior gut assessment does not improve outcomes, and that metabolically potent probiotics will outperform less active strains regardless of species identity.

**Discussion:**

We contend that refocusing from species-level matching to strain-level metabolic potency may offer a more effective and simpler alternative. Future research should prioritize metabolic potency over colony-forming unit counts and reconsider the necessity of routine pre-intervention gut assessment. Our framework does not rely on a single metabolite but embraces diverse mechanisms—proteolysis, carbohydrate fermentation, pH modulation—that collectively restore gut homeostasis.

## 1 Introduction

Current clinical practice often advocates gut microbiome assessment before selecting probiotic species, assuming a ‘test-then-match’ strategy yields better outcomes. Patients pay for a stool test, receive a report listing what is ‘missing’ or ‘imbalanced’, and then take the corresponding probiotic species. This approach is intuitively appealing: if a child lacks *Bifidobacterium longum*, one gives *B. longum*; if *Lactobacillus rhamnosus* is low, one adds *L. rhamnosus*. It follows the same logic as treating a vitamin deficiency—test first, then supplement what is deficient.

Yet the analogy may be flawed. Unlike vitamins, which are chemically defined and function reliably once ingested, probiotic species do not operate like simple replacement therapy. As we will elaborate below, this perspective aligns with—and extends—the recent framework proposed by Sanders and Hill (2025), which concluded that probiotics could act independently of the resident gut microbiota. Systematic reviews and meta-analyses have confirmed the strain-specific efficacy of some probiotics (e.g., *Saccharomyces boulardii* CNCM I-745 and *L. rhamnosus* GG) in specific contexts, such as preventing antibiotic-associated diarrhea (Waitzberg et al., 2024). However, when the same ‘test-then-match’ framework is applied more broadly—particularly in heterogeneous conditions such as autism spectrum disorder (ASD) and attention-deficit/hyperactivity disorder (ADHD)—the results are inconsistent. A 2024 meta-analysis found no significant improvement in total ADHD symptoms (SMD = 0.25; 95% CI [0.07, 0.56]; *p* = 0.12) with probiotics compared to placebo (Liang et al., 2024). Another systematic review similarly concluded that while probiotics may be used as an adjuvant approach for children with ASD and ADHD, their validity and efficacy continue to be debated (Munteanu et al., 2024). Even the clinical utility of routine gut microbiome testing itself remains questionable. At-home tests have not been clinically validated for diagnosis or treatment guidance. An international consensus statement concluded that direct-to-consumer microbiome tests lack proven value in clinical practice and may lead to inappropriate dietary changes, unnecessary purchases, or delayed medical care (Porcari et al., 2025). Independent research has also documented that these tests lack analytical and clinical validity, with potential harms including misleading dietary recommendations and unjustified supplement use (Hoffmann et al., 2025).

We believe that a key reason for these mixed results is not that probiotics are ineffective, but that the field has been asking the wrong question. Instead of asking “which species is missing?,” we should be asking “how do we restore a gut environment that naturally favours health?”

Given the limited predictive value of routine gut microbiome tests and the modest efficacy of precision probiotic interventions in neurodevelopmental disorders, a different approach may be needed. This brings us to our core proposal: prioritize remodelling the gut environment using metabolically potent probiotics, without prior microbiome assessment. Drawing an analogy to an aquarium—conditioning the water first allows native beneficial fish to thrive—we argue that metabolically potent probiotics can modify the gut ecosystem in ways that favour host health. Once the gut microenvironment is conditioned, beneficial bacteria naturally present in food and the environment become more likely to reside in the gut and help the host.

We do not propose that gut microbiome assessment is never useful. Under specific circumstances—such as severe immunocompromised states, suspected inflammatory bowel disease, recurrent *Clostridioides difficile* infection, or research settings—pre-intervention testing may still be appropriate. However, for routine probiotic selection in otherwise healthy individuals—particularly those with neurodevelopmental disorders such as ASD and ADHD, or mood disorders, we hypothesize that it may often be unnecessary. While the framework may have broader applicability beyond these conditions, neurodevelopmental and mood disorders are the primary focus of this hypothesis.

This article is a hypothesis and theory article—not a systematic review or a controlled trial. Its purpose is to propose a new conceptual framework for probiotic selection. We do not claim definitive evidence of causality or efficacy, nor do we intend this as a practice-changing clinical recommendation at this stage. Rather, we aim to stimulate hypothesis-driven research by offering a testable alternative to the prevailing ‘test-then-match’ paradigm.

The novelty of our framework lies not in the recognition that probiotic metabolites matter—a point increasingly acknowledged in the literature—but in three specific propositions that distinguish it from existing function-based probiotic concepts. First, we explicitly deprioritise taxonomic stool profiling as a prerequisite for probiotic selection, questioning the assumption that identifying ‘missing’ species is necessary for therapeutic success. Second, we propose that strain-level metabolic potency—measured through functional outputs such as proteolysis, carbohydrate fermentation, and pH modulation—should take priority over taxonomic matching when selecting probiotics. Third, we hypothesise that *Bifidobacterium* abundance is more often a consequence of a healthy gut environment than a primary cause of it, and that successful environmental remodelling will naturally increase *Bifidobacterium* without direct supplementation. These propositions collectively shift the focus from ‘what is missing’ to ‘how to condition the environment to support what is already present or may naturally arrive’.

To improve transparency, we distinguish between three types of content in this manuscript: (i) published evidence from peer-reviewed preclinical and clinical studies, which is presented as established findings; (ii) logical inference derived from ecological principles and biological plausibility, which is presented as reasoned interpretation; and (iii) unvalidated hypotheses that we propose for testing, which are explicitly identified as hypotheses. This distinction is intended to help readers assess the strength of each claim and to clarify which components of our framework are speculative and which are supported by existing evidence.

Our proposal is consistent with preclinical studies demonstrating the metabolic potency of specific strains of *L. helveticus* and *Lactobacillus fermentum* (e.g., the extensively characterized NS8 and NS9). In the following sections, we elaborate on the rationale for this ‘condition-then-attract’ strategy, in which a metabolically potent probiotic is used to condition the gut environment first, after which native beneficial bacteria are expected to attract and thrive. This paradigm aligns with an emerging emphasis in the field on probiotic metabolites over purely taxonomic composition (Cunningham et al., 2021; Li et al., 2026) and proposes a reframing of post-intervention assessment that focuses on functional markers rather than pre-intervention species mapping. This framework is presented as a testable hypothesis, not as a demonstrated clinical strategy.

**Figure 1:**
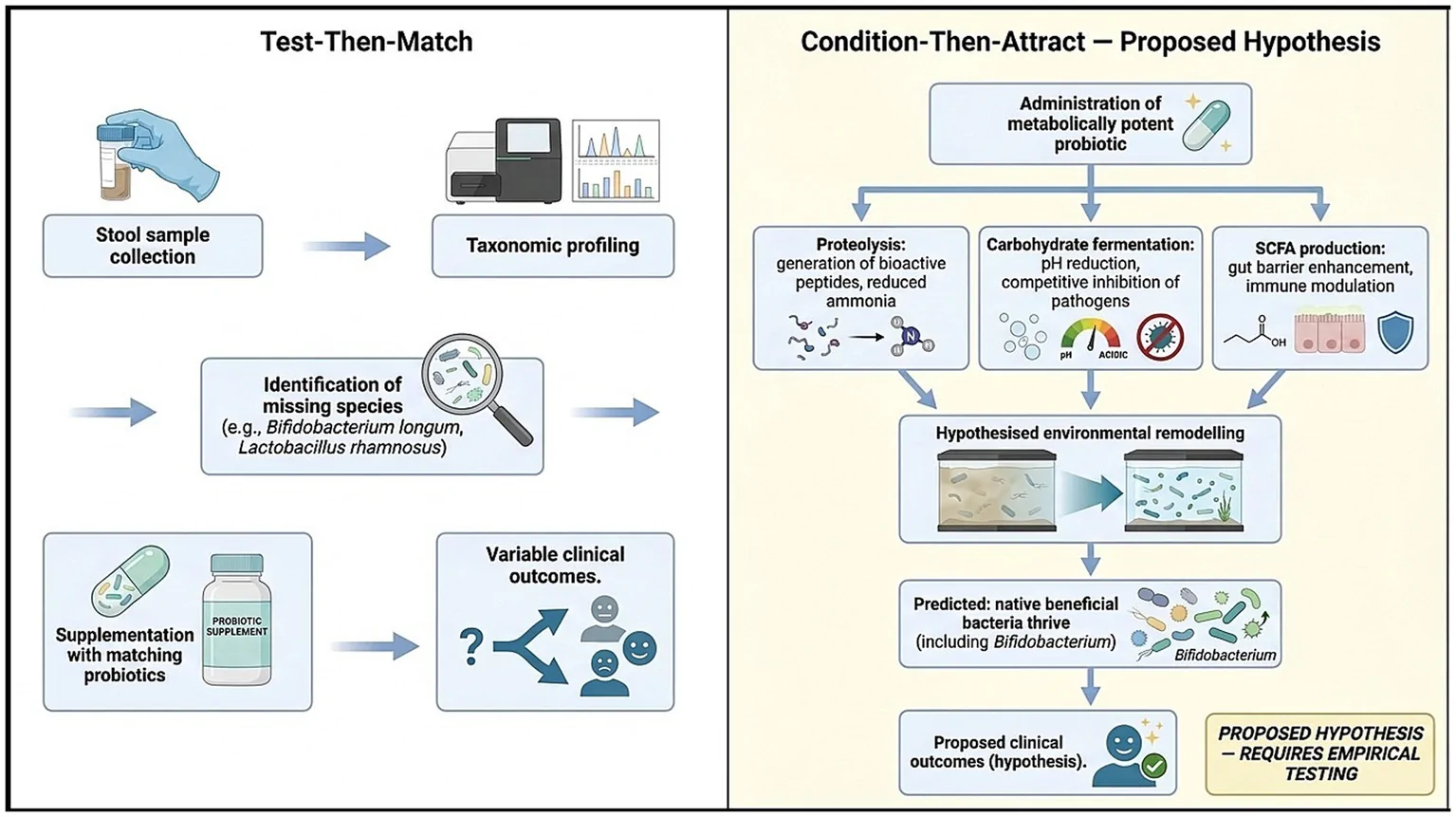
Comparison of the ‘test-then-match’ paradigm and the proposed ‘condition-then-attract’ hypothesis for probiotic selection. (Left) The conventional approach: Stool testing is used to identify missing species, and species-specific probiotics are then administered. (Right) The proposed hypothesis: a metabolically potent probiotic is administered to remodel the gut environment through proteolysis, carbohydrate fermentation, and SCFA production. These activities are hypothesized to remodel the gut environment, after which native beneficial bacteria (including *Bifidobacterium*) are predicted to thrive, leading to clinical improvement. The right panel represents a hypothetical sequence that requires empirical testing. It is not an established outcome.

As shown in Figure 1, the ‘condition-then-attract’ paradigm differs fundamentally from the ‘test-then-match’ approach.

## 2 Rethinking the ‘test-then-match’ paradigm

The idea that one should test the gut microbiome and then supplement the missing species is intuitively appealing. Yet the clinical utility of routine gut microbiome testing for guiding probiotic selection remains unproven (Porcari et al., 2025; Rodriguez et al., 2025). A growing body of literature suggests that while microbiome profiling has research value, its ability to predict individual responses to probiotics is limited (Porcari et al., 2025). To our knowledge, no randomized controlled trial has demonstrated that stool-test-guided probiotic selection leads to better clinical outcomes than empirical selection. While some uncontrolled studies and a crossover trial have examined stool test selected formulations against placebo, these designs do not establish the incremental clinical utility of microbiome guided selection over empirical choice (Porcari et al., 2025).

Even when probiotics are selected based on mechanistic rationales rather than stool testing, the results for neurodevelopmental disorders have been modest. For example, in a six-month double-blind, placebo-controlled trial of a two-strain *L. reuteri* formulation—chosen for its preclinical social-behavior effects—Mazzone et al. (2024) reported significant improvements in social functioning across multiple measures. However, the trial did not detect changes in overall autism severity, restricted and repetitive behaviours, gut microbiota composition, or overall immune activity. This absence of a detectable shift in microbial composition is consistent with the view that probiotic activity may involve functional or localized signalling rather than durable taxonomic restructuring. The positive social outcomes suggest that probiotic effects may be domain-specific rather than broad-spectrum, a distinction that should inform the design of adequately powered future trials. We include this example not as evidence against precision interventions, but to highlight that even mechanistically motivated formulations may produce effects that are not captured by taxonomic profiling, and that larger trials with clinically meaningful adjunctive outcomes and responder subgroup analyses are needed. We acknowledge that some uncontrolled studies and small trials have reported positive outcomes from targeted probiotic interventions. However, the incremental clinical benefit of stool-guided selection over empirical choice remains to be established.

Reflecting the historically inconsistent data surrounding gut-microbiota interventions for neurodevelopmental disorders, a 2024 meta-analysis of probiotics yielded no significant benefit over placebo for total ADHD symptoms (SMD = 0.25; 95% CI [−0.07, 0.56]) (Liang et al., 2024). A separate meta-analysis of eight RCTs for ASD found only a borderline beneficial effect (mean difference −0.21) with high risk of bias in most studies (Kotowska et al., 2024). A systematic review and meta-analysis of probiotic clinical trials in children with ASD similarly concluded that their overall efficacy and behavioural symptoms remain statistically non-significant and open to be debate (He et al., 2023). One interpretation of these mixed findings is not that probiotics are inherently ineffective, but that the species-level ‘test-then-match’ framework may be asking the wrong question. We emphasize that our argument against routine pre-intervention assessment is inferential—based on (i) the inconsistent outcomes of test-guided matching, (ii) the limited predictive value of commercial microbiome tests, and (iii) a plausible alternative mechanism (environmental remodelling). We are not claiming that pre-intervention assessment is never useful, rather, we hypothesise that it may often be unnecessary for routine probiotic selection in otherwise healthy individuals. To avoid misunderstanding, we clarify that our proposal questions the routine use of taxonomic stool profiling—specifically, the identification of ‘missing’ species—as a prerequisite for probiotic selection. We do not propose bypassing all forms of microbiome assessment. Functional measures (e.g., metabolomics, short-chain fatty acid profiling) and clinical assessments (e.g., dietary history, medication exposure, gastrointestinal symptoms) remain valuable for understanding individual variation and interpreting treatment response. However, we hypothesize that the specific step of identifying ‘missing’ species via stool testing may not be necessary for initiating a trial of metabolically potent probiotics in otherwise healthy individuals—particularly those with neurodevelopmental or mood disorders. Consistent with our position in the Introduction, we recognise that pre-intervention gut assessment may still be appropriate in specific contexts—for example, in severe immunocompromised states, suspected inflammatory bowel disease, recurrent *Clostridioides difficile* infection, or research settings where detailed microbiome characterisation is required. Our ‘condition-then-attract’ proposal is not intended to replace these indicated uses, but to question the routine, reflexive ordering of gut tests before every probiotic trial. Therefore, we propose an alternative conceptual framework that prioritises metabolic potency over taxonomic matching. In the following sections, we elaborate on the rationale for this ‘condition-then-attract’ strategy, examining how metabolically potent probiotics might function as environmental remodellers and discuss its testable predictions that arise from this framework.

## 3 Probiotics as environmental remodellers: a hypothesis

We use the term ‘environmental remodellers’ to describe probiotics that alter the gut ecosystem through metabolic activity—such as pH reduction, SCFA production, and proteolysis—rather than through long-term colonization or species replacement.

The gut microbiota is not a static catalogue of species that can be ‘filled in’ like a vitamin deficiency. Instead, it is a dynamic ecosystem shaped by the intestinal environment. A 2024 review highlights that the microbiota acts as a crucial mediator in gut homeostasis and colonization resistance (Chen et al., 2024). Within this ecosystem, beneficial bacteria occupy ecological niches that are determined not by which species are ‘missing’, but by the chemical and physical conditions of the gut—pH, oxygen tension, nutrient availability, and the presence of inhibitory metabolites. For this reason, many ingested probiotics form only a transient microbiome (Derrien and van Hylckama Vlieg, 2015; Lewandowski, 2026; as noted by the International Scientific Association for Probiotics and Prebiotics [ISAPP], Marco, 2019).

A recent perspective by Sanders and Hill (2025) concluded that the official definitions of probiotics and postbiotics do not require that they act through the gut microbiota. In other words, a probiotic can be efficacious by directly engaging with the host—modulating immunity, producing metabolites, or reinforcing the gut barrier—without necessarily changing the composition of the resident microbiome. Empirical support for this view comes from a study using heat-killed probiotics. Zhang et al. (2020) found that oral administration of heat-killed *L. helveticus* NS8, which cannot colonize or metabolize, still significantly altered the hippocampal peptidome in mice, despite causing no substantial change in gut microbiota composition. This indicates that bacterial components (postbiotics) can directly influence the gut-brain axis without any need for live colonization or metabolic activity. While our ‘condition-then-attract’ framework focuses on live, metabolically potent strains to actively remodel the gut environment, the postbiotic effect further reinforces a broader principle: probiotic efficacy does not depend on matching missing species. Direct host–microbe interactions—whether through metabolites, cell wall components, or heat-stable molecules—can be sufficient. We take this argument one step further, as a hypothesis to be tested. In our framework, the probiotic is not simply replacing missing species, nor is it solely replying on interaction with the resident microbiome. Instead, we hypothesize that a metabolically potent probiotic transiently remodels the gut environment—through proteolysis, carbohydrate fermentation, pH modulation, and metabolic production—thereby creating conditions that favour the establishment and activity of native beneficial bacteria (including Bifidobacterium). This is consistent with the fish-tank analogy: the focus is on conditioning the water, not on identifying which fish are missing or adding individual fish species one by one. The probiotic remains a microbial agent. It does not stand outside the ecosystem. The ‘environmental remodeller’ concept is used heuristically to emphasize that the probiotic’s primary contribution may be environmental remodelling rather than long-term engraftment or species replacement. These proposed mechanisms include: (i) rapid degradation of large antigenic proteins (e.g., casein, gluten) into small bioactive peptides (<3,000 Da) and amino acids—a function that has been demonstrated *in vitro* for related strains (Griffiths and Tellez, 2013); (ii) competitive fermentation of simple sugars (fructose, glucose), which may lower pH and suppress opportunistic pathogens through ecological competition (a hypothesis consistent with the principles discussed above); and (iii) production of short chain fatty acids (SCFAs), organic acids, and bacteriocins, which are known to enhance gut barrier function and modulate immunity (Nami et al., 2025) with some effects observed in paediatric populations (Brasiel and Potente Dutra Luquetti, 2025). Together, these mechanisms operate as an integrated cascade. Proteolysis reduces the availability of antigenic proteins and nitrogenous waste products that may disrupt gut homeostasis. Concurrently, carbohydrate fermentation lowers luminal pH, creating conditions that favour acid-tolerant commensals while suppressing pH-sensitive opportunists. The resulting metabolic milieu—enriched in SCFAs, organic acids, and bacteriocins—reinforces gut barrier function, modulates local immune activity, and provides energy substrates for colonocytes. This coordinated sequence of biochemical and ecological changes collectively remodels the gut ecosystem, shifting it from a potentially dysbiotic state towards one that is more receptive to native beneficial bacteria. Collectively, these activities create a habitat that naturally attracts and supports beneficial bacteria from food and the environment. Some of these effects have been observed in paediatric populations. The resident microbiome (including *Bifidobacterium*) would then thrive because of this conditioned environment, not as a prerequisite. Direct evidence for this conditioning role in humans is currently lacking, and mechanistic studies are needed.

The beneficial effects of probiotics are thought to be mediated not by long-term engraftment, but by short-lived metabolites such as SCFAs, bacteriocins, and organic acids that remodel the local environment (He et al., 2024; Ma et al., 2022). SCFAs serve as energy sources for colonic cells, maintain gut barrier integrity, regulate immune responses, and influence systemic metabolic pathways (Ragavan and Hemalatha, 2024; Chen et al., 2024). Thus, measuring changes in the microbiome may not be necessary to assess probiotic efficacy (Sanders and Hill, 2025). If probiotics work primarily by changing the gut environment rather than replacing specific taxa, then pre-intervention assessment of which taxa are missing becomes less relevant—a key inferential step in our argument.

We note that the relative contribution of each metabolite class may vary across strains. We acknowledge that inter-individual variation in baseline gut ecology, diet, and host physiology may also influence probiotic response. However, we hypothesize that the specific step of identifying ‘missing’ species via stool testing may not be necessary for initiating a trial of metabolically potent probiotics in otherwise healthy individuals. For example, *L. helveticus* NS8 possesses a highly active proteolytic system, and its oral administration is heavily implicated in generating functional bioactive peptides from protein degradation, thereby modulating the gut-brain axis and the brain peptidome landscape (Zhang et al., 2020). Consequently, this strain-specific peptidome modulation has been shown to rescue behavioral, cognitive and biochemical aberrations induced by chronic stress, metabolic disorders or genetic predisposition to depression or metabolic toxin-induced neuroinflammation (Alatan et al., 2024; Liang et al., 2015; Luo et al., 2014) In contrast, based on our unpublished *in vitro* screening and subsequent animal studies (including in livestock and poultry), *L. fermentum* NS9 exhibits a strong pH lowering capacity through carbohydrate metabolism, which is consistent with its demonstrated ability to stabilize the gut environment and mitigate antibiotic-induced abnormalities (Wang et al., 2015). Nevertheless, the common principle is that the probiotic’s functional output, rather than its taxonomic identity or long-term colonization, drives environmental change. This ecological perspective directly informs our choice of probiotic strains. Instead of selecting strains for their ability to permanently colonise the gut—a property that most ingested probiotics do not achieve in adults—we prioritise strains that act as transient yet potent environmental remodellers. Two criteria guided our selection: metabolic potency (the strain should produce metabolites that rapidly improve the gut environment) and transient action (the strain does not need to permanently engraft). This ‘condition-then-attract’ logic values what a probiotic *does* over how long it stays. We emphasize that the ‘transient’ nature of our proposed probiotics is not a limitation but a deliberate design feature. First, most orally administered probiotics do not permanently colonize the adult gut under normal conditions. Our framework simply accepts this biological reality (Derrien and van Hylckama Vlieg, 2015). Second, transient colonization avoids potential risks of long-term engraftment, including niche monopolization, unknown long-term metabolic effects, and horizontal gene transfer. Third, the beneficial action of a probiotic is primarily mediated by its metabolites, which are produced during even a brief intestinal passage. Thus, a ‘condition-then-attract’ strategy deliberately favours metabolically potent, transient strains that condition the environment and then exit, allowing native beneficial bacteria to recolonize naturally.

An important caveat concerns the durability of the proposed environmental effects. The administered probiotic is transient. Therefore, the changes it produces (e.g., pH reduction, SCFA elevation, redox modulation) may also be transient. The terms “restoration” and ‘environmental remodelling’ could imply a durable ecological transition that may not occur without continued probiotic administration. We therefore distinguish between on-treatment functional effects, which represent temporary support, and sustained post-intervention ecological change, which would require follow-up measurements after an appropriate washout period. If the effects require continued administration, the intervention may represent functional conditioning rather than permanent habitat restoration—a distinction that should be tested empirically in future trials. We have therefore used the term ‘condition’ rather than ‘restore’ throughout this framework to reflect this distinction.

We emphasise that this framework remains a hypothesis. Its validity will need to be tested through direct comparisons of ‘test-then-match’ versus ‘condition-then-attract’ strategies in controlled trials. Having outlined the conceptual basis for the framework, we next turn to specific examples of metabolically potent probiotic strains that illustrate how the principle of metabolic potency can be operationalised in practice.

We note that the probiotic remains a microbial agent. It does not stand outside the ecosystem. The ‘ecosystem engineer’ concept*—*borrowed from ecology*—*is used heuristically to emphasise that the probiotic’s primary contribution may be environmental remodelling rather than long-term engraftment.

## 4 Metabolic potency matters more than taxonomic composition

To illustrate the principle, we draw on several well-characterized probiotic strains that have been studied across diverse preclinical models. The purpose is not to promote specific commercial products, but to provide concrete examples of how the ‘condition-then-attract’ framework could be operationalised. Any strain that meets the criteria of high metabolic activity and transient gut persistence could serve the same role—irrespective of its species name. By ‘metabolic potency’ we refer to a strain’s capacity to efficiently transform dietary components (proteins, carbohydrates, lipids) into beneficial bioactive metabolites. This includes: (i) rapid proteolysis—breaking down large antigenic proteins (e.g., casein, gluten, whey, soy) into small bioactive peptides (<3,000 Da) and amino acids (Griffiths and Tellez, 2013; Skrzypczak et al., 2017), thereby reducing the production of neurotoxic nitrogen- and Sulphur-containing compounds (ammonia, mercaptans); (ii) competitive carbohydrate fermentation–rapidly consuming simple sugars (fructose, glucose) to lower pH and suppress opportunistic pathogens (see Louca et al., 2018 for a broader review of functional decoupling); and (iii) short-chain fatty acid (SCFA) production, which supports gut barrier integrity and immune regulation (Ragavan and Hemalatha, 2024). To make this concept operationally useful for strain selection, we propose the following measurable criteria for identifying metabolically potent probiotic candidates:

- Metabolite production: the strain should generate quantifiable beneficial metabolites *in vitro*, including short-chain fatty acids (e.g., butyrate, propionate, acetate ≥2 mM in standard culture), organic acids (e.g., lactate ≥10 mM), and/or bioactive peptides from protein substrates;
- Functional activity: the strain should demonstrate functional effects such as pH reduction (≥0.5 pH units within 12 h of fermentation), proteolytic activity (≥50% hydrolysis of casein or gluten within 24 h), or inhibition of enteric pathogens (≥50% reduction in growth of indicator strains);
- Transient gut persistence: the strain should be detectable in faecal samples during administration but not after a washout period, consistent with the transient nature of probiotic action.

These criteria are intended as screening-level attributes to identify candidate strains for further investigation. They do not constitute evidence of clinical efficacy, which requires additional validation through indication-specific trials, dose-finding studies, and assessment of host and dietary context.

We use the term ‘metabolic potency’ to refer to the intrinsic capacity of a strain to produce beneficial metabolites under defined conditions. However, we acknowledge that the actual metabolic function of a probiotic *in vivo* (its ‘metabolic potential’) may vary across individuals due to differences in diet, host physiology, and luminal environment. This distinction should be considered when translating *in vitro* screening results to clinical application.

Different strains may excel in different aspects. The common principle is measurable functional output that remodels the gut environment. This can be assessed through vitro assays (e.g., proteolytic activity, sugar fermentation rate, SCFA quantification, pH reduction) before clinical use. Some strains may excel in one or more of these dimensions. The examples below focus on proteolysis (*L. helveticus* NS8) and carbohydrate fermentation (*L. fermentum* NS9), which represent two major pathways of environmental remodelling. Short-chain fatty acid production, while also a marker of metabolic potency, is not the primary mechanism for these specific strains. We acknowledge that these criteria are screening-level attributes to identify candidate strains for further investigation. They do not constitute evidence of clinical efficacy, which requires additional validation through indication-specific trials, dose-finding studies, and assessment of host and dietary context.

Key functional attributes of metabolically potent probiotics include:

- Production of short-chain fatty acids (SCFAs) in measurable amounts
- Ability to lower pH in standard culture media
- Demonstrated inhibition of enteric pathogens via organic acids or bacteriocins
- Rapid degradation of large proteins (casein, gluten, whey, soy) into small bioactive peptides (<3,000 Da)
- Strong competitive consumption of simple sugars (fructose, glucose) to inhibit pathogenic bacteria and fungi

*Example 1: Strains of L. helveticus and L. fermentum.* Some strains of *L. helveticus* and *L. fermentum*, such as the extensively studied NS8 and NS9, exhibit high metabolic activity and demonstrate only moderate survival in simulated gastrointestinal conditions, suggesting they do not rely on permanent colonization. Notably, *L. helveticus* is considered the most proteolytically active species among lactobacilli, capable of efficiently degrading casein, gluten, whey, soy, and other proteins (Griffiths and Tellez, 2013; Pescuma et al., 2013; Sadat-Mekmene et al., 2011; Sasaki et al., 1995). NS8 was selected from this species as the strongest and fastest protein-metabolizing strain. Our unpublished *in vitro* observations indicate that its primary active metabolites are small bioactive peptides.

*Protein metabolism (NS8)*. NS8 rapidly digests large, complex protein molecules into small bioactive peptides (below 3,000 Daltons) and amino acids—forms that cells can directly utilise. This process markedly reduces the production of nitrogen-containing compounds (e.g., ammonia) and volatile sulphur-containing substances that arise from incomplete protein breakdown. These metabolites are known to affect the nervous, endocrine, and immune systems, and have been linked to cognitive impairment, memory loss, and behavioural abnormalities. In a rat model of chronic hyperammonaemia (a model of hepatic encephalopathy), NS8 supplementation reduced depressive- and anxiety-like behaviours, improved cognitive function, attenuated neuroinflammation, and restored normal tryptophan metabolism (Luo et al., 2014). In a rat model of endogenous depression, NS8 improved depressive-like behaviours, alleviated serotonergic system abnormalities, and reduced hypothalamic corticotrophin-releasing hormone (CRH) expression, without strong changes in SCFAs (Alatan et al., 2024). A peptidomes study on *L. helveticus* NS8 further demonstrated that oral probiotic administration remodelled the brain peptidome, which included modulating CRH and other neuropeptides within the hypothalamus to alleviate HPA axis hyperactivity (Zhang et al., 2020). A similar mechanism may apply to NS8’s downstream therapeutic pathways, which may be facilitated by highly active proteolytic system inherent to the *L. helveticus* species (Griffiths and Tellez, 2013). This protein-metabolising capacity may explain NS8’s anti-inflammatory and neuroprotective effects in the gut, as well as its neuroprotective benefits, including the upregulated expression of brain-derived neurotrophic factor (BDNF) (Liang et al., 2015) and reduced ammonia-induced brain damage (Luo et al., 2014).

*Carbohydrate metabolism (NS9)*. Simple sugars such as fructose and glucose can promote the overgrowth of harmful intestinal microorganisms, including Gram-negative bacteria like *Klebsiella* (Xue et al., 2023), Gram-positive anaerobes (Hecht et al., 2024), and opportunistic fungi such as *Candida* (Jawhara, 2023). The consumption of sugar-sweetened beverages is positively associated with ADHD symptoms in children (Farsad-Naeimi et al., 2020). Additionally, excessive sugar intake has been linked to systemic inflammation (Bujtor et al., 2021) and pediatric atopic diseases, such as asthma and allergic rhinitis (Prendergast and Barański, 2026) In our unpublished *in vitro* screening and subsequent animal studies (including in livestock and poultry), *L. fermentum* NS9 has an exceptionally strong ability to metabolise simple sugars. It rapidly competes for these sugars, lowering local pH and thereby suppressing the growth and metabolite production of harmful microbes. In a preclinical study, NS9 normalised antibiotic-disrupted gut microbiota, reduced colonic inflammation, and restored cognitive function (Wang et al., 2015). In a model of retinal degeneration, NS9 combined with aronia anthocyanidin extract provided better protection against oxidative stress than the extract alone, with increased antioxidant enzyme activities and upregulation of crystallin proteins (Xing et al., 2023). This sugar-consuming, pH-lowering property positions NS9 as an effective ‘environmental remodeller’ that limits the proliferation of opportunistic pathogens.

*Additional lipid metabolism*. Beyond protein and carbohydrate metabolism, certain NS strains (e.g., *L. plantarum* NS5) have been shown to improve lipid metabolism, including reducing triglycerides and cholesterol in high-cholesterol-fed rats (Hu et al., 2013). This broader metabolic versatility underscores the principle that selecting probiotics for functional metabolic output—rather than taxonomic matching—may have wide-ranging health benefits.

NS5, NS8 and NS9 are used as illustrative cases because they have been extensively characterized. The framework does not require their use.

*Example 2: Other metabolically potent strains.* The same principle can be extended to strains from other species. For instance, specific strains of *Lactiplantibacillus plantarum* have been shown to increase short-chain fatty acid (SCFA) production and enrich beneficial taxa such as *Bifidobacterium* in animal models (Ma et al., 2022). Certain *Lacticaseibacillus rhamnosus* strains exhibit diverse metabolite profiles (e.g., inosine, N-acetylisoleucine, phenyllactate) and robust metabolic activity in complex fermentation substrates (Chamberlain et al., 2022). Even among *Bifidobacterium* species—which we have re-framed as potential ‘consequences’ of gut health—some strains (e.g., *Bifidobacterium breve* BBr60) can significantly elevate gut SCFA levels (An et al., 2026). Thus, a strain’s efficacy in remodelling the gut environment may depend less on its taxonomic name than on its functional metabolic output.

To facilitate comparison, the strains discussed above are summarised in Table 1.

**Table 1:** Examples of metabolically potent probiotic strains and their primary metabolic activities.

Strain	Primary metabolic activity	Key metabolites	Preclinical evidence (examples)
*L. helveticus* NS8	Proteolysis	Bioactive peptides, reduced ammonia	Alatan et al. (2024), Liang et al. (2015), Luo et al. (2014), Zhang et al. (2020)
*L. fermentum* NS9	Carbohydrate fermentation (simple sugars)	Lactic acid, pH reduction, SCFAs	Wang et al. (2015), Xing et al. (2023)
*L. plantarum* NS5	Lipid metabolism	Reduced triglycerides, cholesterol	Hu et al. (2013)
*L. plantarum* (other strains)	SCFA production	Butyrate, propionate, acetate	Ma et al. (2022)
*L. rhamnosus* (other strains)	Diverse metabolite production	Inosine, phenyllactate	Chamberlain et al. (2022)
*B. breve* BBr60	SCFA production	Butyrate, propionate, acetate	An et al. (2026)

*From species to function*. A growing emphasis in the field is moving away from species-level identification and toward functional (metabolite-based) assessment of probiotics (Li et al., 2026). Indeed, the beneficial effects of probiotics are largely mediated by SCFAs, bacteriocins, and organic acids that remodel the local environment (He et al., 2024; Ma et al., 2022). These metabolites, rather than the bacterial cells themselves, are primary drivers of improved gut barrier function, immune modulation, and systemic effects (Ragavan and Hemalatha, 2024). Therefore, we argue that screening probiotics for metabolic potency—their capacity to produce beneficial metabolites—should take priority over counting colony-forming units or matching species to a pre-intervention test. This shift from taxonomic composition to functional capacity is a testable hypothesis that, if confirmed, could help move the field beyond the inconsistent results of the ‘test-then-match’ approach. We note that different metabolically potent strains may achieve environmental remodelling through distinct primary mechanisms—protein degradation (e.g., *L. helveticus*), simple carbohydrate fermentation (e.g., *L. fermentum*), or SCFA production (e.g., certain *L. plantarum* and *Bifidobacterium* strains). The common principle is functional metabolic output that improves gut ecology, not a single pathway.

*Caveat*. We acknowledge that the above examples are drawn primarily from preclinical studies. Direct evidence that selecting probiotics based on metabolic potency improves clinical outcomes compared to species-level matching is still lacking, and this framework therefore requires testing in future randomised controlled trials.

If metabolically potent probiotics can indeed remodel the gut environment through proteolysis and carbohydrate fermentation, an important question arises: what happens to the resident microbial community—particularly *Bifidobacterium*, a genus often used as a marker of gut health? The next section addresses this question by examining whether Bifidobacterium should be viewed as a cause or a consequence of gut health.

## 5 Rethinking *Bifidobacteria*: consequence or cause?

We hypothesise that if metabolically potent lactobacilli such as NS8 and NS9 can transiently modify gut redox potential and pH through proteolysis and carbohydrate fermentation, then strict anaerobes like *Bifidobacterium* would be expected to thrive as a result of this environmental remodelling—a prediction that requires empirical testing. *Bifidobacterium* species are predominantly anaerobic commensals highly abundant in the distal colon, although some may possess specific genetic adaptations allowing them to survive in distinct ecological niches, including the human oral cavity and insect gastrointestinal tracts. They also require a low redox environment for optimal growth and have developed mechanisms to tolerate acidic conditions (O'Callaghan and van Sinderen, 2016). In contrast, most lactic acid bacteria (including lactobacilli) are facultative anaerobes capable of surviving transit and exerting functional effects across distinct niches of the gastrointestinal tract-from the oral cavity to the colon—even if many function as transient (allochthonous) rather than permanent residents (Walter, 2008). This ecological difference explains why metabolically potent lactobacilli can serve as early-acting environmental remodellers that condition the gut environment, after which *Bifidobacterium* naturally thrives consequently. A systematic review of *Bifidobacterium* in liver diseases found that low *Bifidobacterium* levels are associated with adverse clinical outcomes, but interventional studies that improved clinical outcomes also increased *Bifidobacterium* abundance (Hizo and Rampelotto, 2023). This raises the possibility that, in many circumstances, *Bifidobacterium* may be a consequence of a healthy gut environment rather than a primary cause. In preterm infants, early *Bifidobacterium* deficiency appears to be a negative biomarker of adverse neurological outcomes (Beghetti et al., 2023). This does not prove that supplementing *Bifidobacterium* would correct the deficiency. Instead, it suggests that environmental remodelling that naturally increases *Bifidobacterium* may also improve clinical outcomes. We propose the following hypothesis, consistent with our ‘condition-then-attract’ framework: a course of metabolically potent, transient probiotics can transiently condition the gut environment (lowered redox potential, increased SCFAs, reduced inflammation), which in turn creates favourable conditions for *Bifidobacterium* to thrive—without the need to test for or directly supplement it. If this hypothesis is confirmed, then *Bifidobacterium* abundance would serve as a secondary biomarker of successful environmental remodelling, not the primary therapeutic target.

We do not claim that *Bifidobacterium* is never causally important. In specific contexts—such as breastfed infants benefiting from human milk oligosaccharides, or certain metabolic pathways where *Bifidobacterium* directly produces beneficial metabolites—direct supplementation may still provide added value. However, for routine probiotic selection in otherwise healthy individuals with neurodevelopmental or mood disorders, we hypothesise that upstream environmental remodelling may be more efficient than species-level matching. This distinction is testable: a randomised trial could compare direct *Bifidobacterium* supplementation versus ‘condition-then-attract’ (using metabolically potent lactobacilli) and measure both clinical outcomes and subsequent *Bifidobacterium* levels.

This view shifts the focus from ‘replacing missing species’ to ‘restoring the habitat that allows them to flourish’. We hypothesize that a successful ‘condition-then-attract’ intervention will naturally increase Bifidobacterium as a result, without requiring direct supplementation. In the following Discussion, we consider the broader implications of this framework for clinical practice and research, acknowledge its limitations, and outline a roadmap for empirical validation.

## 6 Discussion

### 6.1 General interpretation and implications

Our ‘condition-then-attract’ paradigm has several hypothetical implications for practice and research. First, we hypothesise that, in some contexts, clinicians and patients may not always need to wait for gut microbiome assessment before initiating a trial of metabolically potent probiotics. Early intervention in neurodevelopmental disorders is widely recognised as important for improving long-term outcomes. However, whether probiotic intervention specifically should be initiated without delay is a hypothesis that requires testing. This remains a hypothesis, not a clinical recommendation. Second, our proposal shifts the focus from species-level matching to strain-level metabolic potency, suggesting that probiotic screening could prioritise functional assays (e.g., short-chain fatty acid production, anti-inflammatory cytokine induction, pathogen inhibition) over taxonomic identification alone. This suggestion is hypothesis-driven and awaits empirical validation. Third, observational studies have suggested that free-sugar restriction might be associated with improved outcomes in some populations, whereas gluten- and casein-free diets have not consistently shown additional benefit (Piwowarczyk et al., 2018). However, the intervention combined probiotics with dietary advice; therefore, the observed effects cannot be attributed solely to probiotics. Diet itself is a powerful environmental remodeller, and part of the ‘conditioning’ may be mediated by reduced fermentable sugars.

On a mechanistic level, beyond protein and carbohydrate metabolism, other NS strains such as *L. plantarum* NS5 have demonstrated lipid-lowering effects in animal models (Hu et al., 2013), further illustrating that metabolic potency can be expressed through different substrates.

### 6.2 Limitations

Several limitations should be acknowledged. The preclinical studies cited were conducted in animal models and do not directly mirror human ASD/ADHD. While these models provide valuable mechanistic insights—particularly regarding proteolytic activity, carbohydrate fermentation, and gut-brain signalling—they have inherent limitations. Rodent models do not fully recapitulate the complexity of human neurodevelopmental disorders, nor do they capture heterogeneity in human diet, genetics, and microbiome composition. Furthermore, the controlled laboratory conditions under which these studies were conducted may not reflect the variability of real-world human environments. Translating findings from animal models to clinical practice therefore requires caution, and the predictions derived from this framework must be tested directly in human trials.

Third, the proposal that pre-intervention gut assessment is unnecessary requires direct testing in randomised controlled trials comparing ‘test-then-match’ versus ‘condition-then-attract’ sequences.

### 6.3 Testable predictions and future directions

From this hypothesis, we derive several directly testable predictions. The following are derived from our hypotheses and await empirical testing. They are not conclusions:

- A randomised controlled trial comparing ‘test-then-match’ versus ‘condition-then-attract’ strategies (using the same metabolically potent strains in both arms) could directly test whether prior gut assessment improves outcomes.
- Metabolically potent probiotics—whether through rapid proteolysis (generating bioactive peptides), competitive carbohydrate fermentation (lowering pH and suppressing pathogens), or short-chain fatty acid production—will outperform less active strains regardless of species identity.
- If *Bifidobacterium* abundance is indeed a consequence of gut health, then successful ‘condition-then-attract’ intervention will naturally increase *Bifidobacterium* levels without direct supplementation.
- The role of free-sugar restriction as an adjunct to metabolically potent probiotics warrants further investigation, ideally under controlled dietary protocols.

Beyond these specific predictions, larger, double-blind, placebo-controlled trials are needed to confirm the efficacy of this approach and to distinguish the effects of probiotics from those of dietary changes and other concurrent interventions. A key unresolved question is the potential interaction between probiotic supplementation and dietary factors*—*particularly free-sugar intake*—*which may confound or enhance probiotic effects. *This question arises from general nutritional science, not from our own data, and requires systematic investigation under controlled dietary protocols*. Future trials should include a control arm with dietary advice alone (or placebo probiotics) to isolate the probiotic effect as well as baseline and serial measurements of the hypothesized environmental targets (e.g., pH, SCFAs, inflammatory markers), Bifidobacterium abundance, clinical response, and post-intervention persistence after a washout period to distinguish on-treatment effects from sustained ecological change.

Beyond these specific trial design recommendations, a comprehensive validation roadmap should include: (i) randomised controlled trials with active comparator arms to compare ‘condition-then-attract’ against both placebo and empirical probiotic selection; (ii) metabolomic analyses to confirm that the predicted metabolic changes (e.g., SCFA elevation, pH reduction) occur in response to intervention; (iii) microbiome functional profiling (e.g., metatranscriptomics or metaproteomics) to assess whether the probiotic alters the functional capacity of the resident microbial community; (iv) strain comparison studies to test whether metabolically potent strains outperform less active strains from the same species; and (v) longitudinal follow-up after washout to determine whether any observed ecological or clinical effects are sustained or require continued administration. These studies, conducted in sequence, would provide the empirical foundation needed to move the ‘condition-then-attract’ hypothesis from a conceptual framework to an evidence-based strategy.

### 6.4 Concluding remarks

Current clinical practice often assumes that gut microbiome assessment should precede probiotic selection. We have argued that this ‘test-then-match’ framework may be based on an oversimplified analogy to vitamin deficiency. Instead, we propose a ‘condition-then-attract’ hypothesis: metabolically potent probiotics could be used to condition the gut environment first, without prior assessment. Once the gut ecosystem is conditioned, beneficial bacteria naturally present in food and the environment may colonise and support host health.

The novelty of this hypothesis lies in three propositions: (i) deprioritizing taxonomic stool profiling as a prerequisite for probiotic selection; (ii) prioritising strain-level metabolic potency—measured through functional outputs such as proteolysis, carbohydrate fermentation, and pH modulation—over species-level matching; and (iii) viewing Bifidobacterium abundance as a consequence, rather than a cause, of a healthy gut environment.

Preclinical studies of selected metabolically potent *Lactobacillus* strains are consistent with this paradigm. However, these findings are not conclusive The framework generates several directly testable predictions—including that metabolically potent strains will outperform less active strains regardless of species identity, and that successful intervention will naturally increase Bifidobacterium without direct supplementation—but these predictions require empirical validation in adequately powered controlled trials., If confirmed, this framework could shift the focus of probiotic research and clinical practice from taxonomic matching to functional conditioning, potentially simplifying selection and improving outcomes by prioritising what probiotics do over which species they are. Until such evidence emerges, our viewpoint should be seen as a conceptual reframing to stimulate debate and hypothesis-driven investigation, rather than a practice-changing clinical recommendation.

## Data Availability

The original contributions presented in the study are included in the article/Supplementary material, further inquiries can be directed to the corresponding authors.
