# Supplementary material for: Condition the water before adding the fish: a hypothesis on probiotic selection without prior gut assessment
Source: Front Microbiol. 2026 Aug 26;17:1931519. doi: 10.3389/fmicb.2026.1931519 (PMC13553955; doi:10.3389/fmicb.2026.1931519)
Supplement: Supplementary file 1 [file Supplementary_file_1.docx]

# Supplementary Material S1

**Title:** Artificial Intelligent (A.I.) prompts and outputs for Figure 1 preparation

## 1. Description:

This supplementary file documents the use of FigureLabs, accessed via <https://chat.figurelabs.ai> on 4 August 2026) to assist in the visual preparation of Figure 1 of the manuscript titled ‘Condition the Water Before Adding the Fish: A Viewpoint on Probiotic Selection Without Prior Gut Assessment’.

The AI tool was used to generate a split‑scene conceptual illustration comparing the ‘test‑then‑match’ paradigm (left) and the proposed ‘condition-then‑attract’ paradigm (right), using an aquarium analogy. The figure was developed through iterative refinement. The full prompt and the corresponding outputs are documented below. The final figure was reviewed by the authors to ensure scientific accuracy, clarity, and consistency with the manuscript’s conceptual framework.1. Prompt Sequence Provided to Gemini

Prompt 1 (Initial request):

**Left panel (Test-Then-Match):**

“Stool sample collection” → “Taxonomic profiling” → “Identification of missing species (e.g., *Bifidobacterium longum, Lactobacillus rhamnosus*)” → “Supplementation with matching probiotics” → “Variable clinical outcomes.”

**Right panel (Condition-Then-Attract – Proposed Hypothesis):**

“Administration of metabolically potent probiotic” → three parallel pathways:
• “Proteolysis: generation of bioactive peptides, reduced ammonia”
• “Carbohydrate fermentation: pH reduction, competitive inhibition of pathogens”
• “SCFA production: gut barrier enhancement, immune modulation”
These converge to “Hypothesised environmental remodelling” → “Predicted: native beneficial bacteria thrive (including Bifidobacterium)” → “Proposed clinical outcomes (hypothesis).”

**Top or bottom annotation (added to the right panel):**

“PROPOSED HYPOTHESIS — REQUIRES EMPIRICAL TESTING”

## 2. Output Received from FigureLabs:

The AI generated image outputs after inputting the prompt. The output was iterative visual drafts that progressively refined the split‑scene illustration. This served as Figure 1 in the manuscript as follows:

## 3. Author Modifications to the AI Output

1. **Adding Frame** – Figure 1 is the result of the authors’ subsequent manual adjustment after adding the black picture frame by authors as the background colour in the left part is white.
2. **Ensured scientific accuracy**: Verified that the visual representation accurately reflected the conceptual distinction between the two paradigms as described in the manuscript.

The final figure is entirely consistent with the authors’ conceptual design and scientific intent. The AI was used solely as a visualisation tool. All scientific content, interpretations, and conclusions remain the sole responsibility of the authors.

## 4. Declaration of AI Use

The authors acknowledge the use of A.I. (FigureLabs accessed via <https://chat.figurelabs.ai> on 4 August 2026) to assist in the visual preparation of Figure 1. The AI tool was used to generate the conceptual diagram comparing the ‘test‑then‑match’ and ‘condition‑then‑attract’ paradigms, based on the authors’ own design and scientific input. All scientific content, interpretations, and conclusions are solely the work of the authors. The prompts provided to the AI and the outputs received are documented above.
